# Digitalis in Pneumonia

**Published:** 1891-10-17

**Authors:** 


### THERAPEUTICS.

### DIGITALIS IN PNEUMONIA.

We have previously incidentally referred to the employment of digitalis in pneumonia, but recently published results would seem to show that it is by the employment of large doses that we get the beneficial effects of this drug in pneumonia.

Since 1882 Dr. Petrescu, Professor of Therapeutics at Bucharest, has continually prescribed digitalis in the enormous doses of 4 to 10 grammes daily, in the form of infusion, in the treatment of pneumonia. He affirms that this treatment gives far better results than any other treatment (2·06 per cent. mortality); that it is free from danger, and never gives rise to any poisonous symptoms, such as vomiting, diarrhœa, intermittent or irregular pulse, collapse, &c. In spite of these statements, and of reports of his method made to the Academy of Medicine at Paris, to the International Congress at Berlin, and to the Therapeutical Congress of 1889, M. Petrescu has never had, up to the present, any imitators, a fact easy of comprehension.

It is, however, well to know the facts brought before the Therapeutical Society by M. Huchard, and the result of Dr. Bardet's experience; these two authors, basing their opinions, the one upon clinical observation, the other upon its physiological action upon animals, have affirmed that digitalis ought to be employed in much more considerable doses than those generally indicated in formulæ.

Quite recently an Austrian army physician, M. Fikl, who has had the courage to try digitalis in large doses in pneumonia, has come to the same conclusions, in the main, as M. Petrescu. M. Fikl was induced to try the method on account of the bad results attending the usual modes of treatment of pneumonia, by which he had lost seven patients out of forty-four, *i.e.*, a mortality of 15·8 per cent. He began to employ digitalis, not without fear, but the results have been so encouraging from the commencement that he has not hesitated to continue the method. Up to the present he has collected already sixty-one cases of pneumonia (of which forty-seven were fibrinous and fourteen lobular), treated by digitalis in large doses. Out of all these only one patient died, making about an average mortality of 1·65 per cent., a striking contrast to the figure in the preceding statistics of the same observer. M. Fikl administers to his patient every twenty-four hours up to the time of defervescence, an infusion of three grammes of digitalis to 200 grammes of water. Even with these doses, small compared with the dose given by M. Petrescu, he has seen some disagreeable symptoms arise; a moderate degree of collapse in the case of two patients, vomiting in twelve cases, intermittence of the pulse in four cases, and sometimes diarrhœa. These accidents, which each time disappeared without disaster, had only necessitated the cessation of the remedy for a short space of time, and have been amply compensated by the excellent effects of the treatment.

By the observations of M. Fikl, the duration of the pneumonia was not perceptibly shortened, but the disease revealed, under the influence of this drug, a mild character. About the second, third, or fifth day at the latest, the temperature begins to abate gradually, the pulse becomes less and less frequent, and the general condition ameliorates. Generally the pneumonia acquires, by the action of digitalis, the tendency to end by lysis rather than by the usual method of crisis. After complete defervescence the pulse often falls to 60 or 50 pulsations the minute. In some cases it has been 38 and even 34 a minute. This bradycardia has never been followed by dangerous symptoms, and completely disappeared in a few days.

Like M. Petrescu, M. Fikl has observed a singularly rapid convalescence in pneumonia treated by digitalis. M. Fikl believes that these observations may fairly lead him to conclude that not only is the method of M. Petrescu an excellent therapeutical acquisition, but that it is perhaps the best of all methods of treating pneumonia. At any rate, one of the special results of these observations has been that it is clear that the therapeutics of digitalis are still little known, above all as regards the question of dose. (*Les Nouveaux Remèdes*, 1891, No. 18.)

